# 275. Genotype-to-Phenotype Agreement of Antimicrobial Resistance in *Escherichia coli*

**DOI:** 10.1093/ofid/ofad500.347

**Published:** 2023-11-27

**Authors:** Munok Hwang, Hosoon Choi, Thanuri Navarathna, Katherine Boykin, Brandon Corona, Sorabh Dhar, Curtis Donskey, Jennifer Cadnum, Piyali Chatterjee, Keith S Kaye, Chetan Jinadatha

**Affiliations:** Central Texas Veterans Health Care System, Temple, Texas; Central Texas Veterans Health Care System, Temple, Texas; Central Texas Veterans Health Care System, Temple, Texas; Central Texas Veterans Research Foundation, Temple, Texas; Central Texas Veterans Health Care System, Temple, Texas; Wayne State University/Detroit Medical Center, John Dingell VAMC, Detroit, Michigan; Cleveland VA Hospital, Cleveland, Ohio; Northeast Ohio VA Medical Center, Cleveland, Ohio; Central Texas Veterans Health Care System, Temple, Texas; Rutgers Robert Wood Johnson Medical School; Central Texas Veterans Health Care System, Temple, Texas

## Abstract

**Background:**

Most *Escherichia coli* reside as commensal intestinal microflora without harming human health. But some *E. coli* strains are pathogenic and can cause deadly infections. Antimicrobial resistant *E. coli* is associated with more than 1.5 million deaths globally. Here, we studied the prevalence of antimicrobial resistance in *E. coli* isolates from two cities based on the antimicrobial(AMR) genes and MIC values. By correlating genotypic and phenotypic antimicrobial resistance, we also examined the performance of whole genome sequencing (WGS) based prediction of phenotypic resistance.

**Methods:**

A total 109 *E. coli* isolates from Detroit, MI and Cleveland, OH hospitals underwent WGS. Antibiotic susceptibility testing (AST) was performed using the gram-negative AST cards on the VITEK 2 system. WGS was performed with Nextseq 550 using Nextera flex kits. After *de novo* assembly, AMR genes were identified using AMR gene database, ResFinder.

**Results:**

The resistance to beta-lactam antibiotics including 1^st^ and 3^rd^ generation cephalosporins were very high among the *E. coli* isolates. They were susceptible to penicillin combinations and 2^nd^ generation cephalosporins. While resistance to quinolone antibiotics was high, resistance to aminoglycoside, tetracycline, and sulfonamide/trimethoprim were relatively low (Figure 1). As shown in Table 1, WGS-based method accurately predicted the majority of antimicrobial resistance phenotypes with accuracy of 0.89 ∼ 1.0. However, for cephalothin (0.36), piperacillin/tazobactam (0.69), and amikacin (0.63), the prediction accuracy was low which could potentially lead to false predictions of antimicrobial resistance. Extended Spectrum Beta-Lactamase (ESBL) genes such as *bla*CTX-M, *bla*OXA, and *bla*TEM were abundant, though subtle difference in the prevalence of AMR genes between two cities was observed (Figure 2).

**Figure 1.**
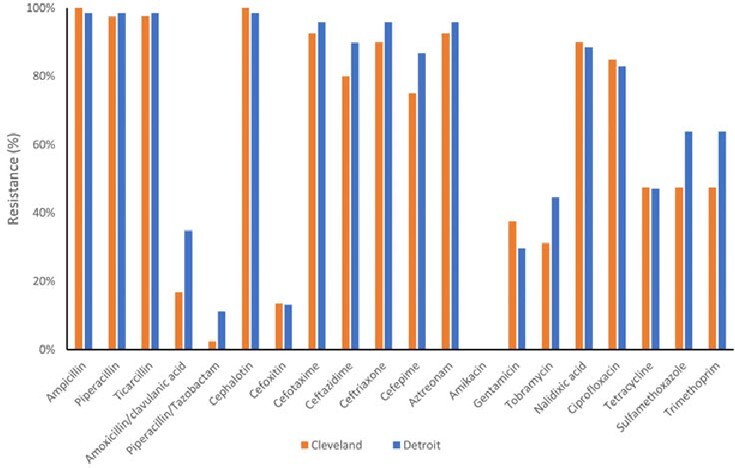
The prevalence of antimicrobial resistance to various antibiotics in E. coli isolates from Detroit, MI and Cleveland, OH.

**Figure:**
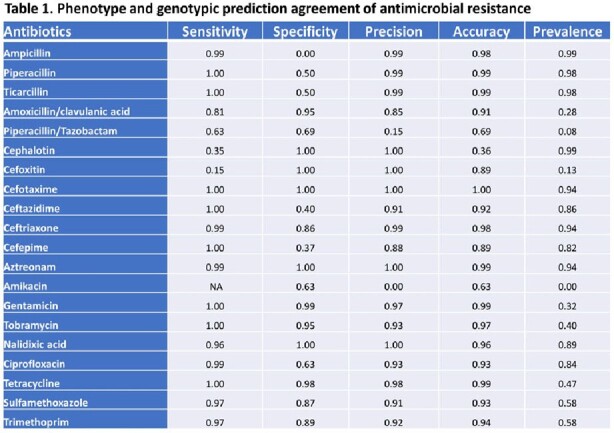

**Figure 2.**
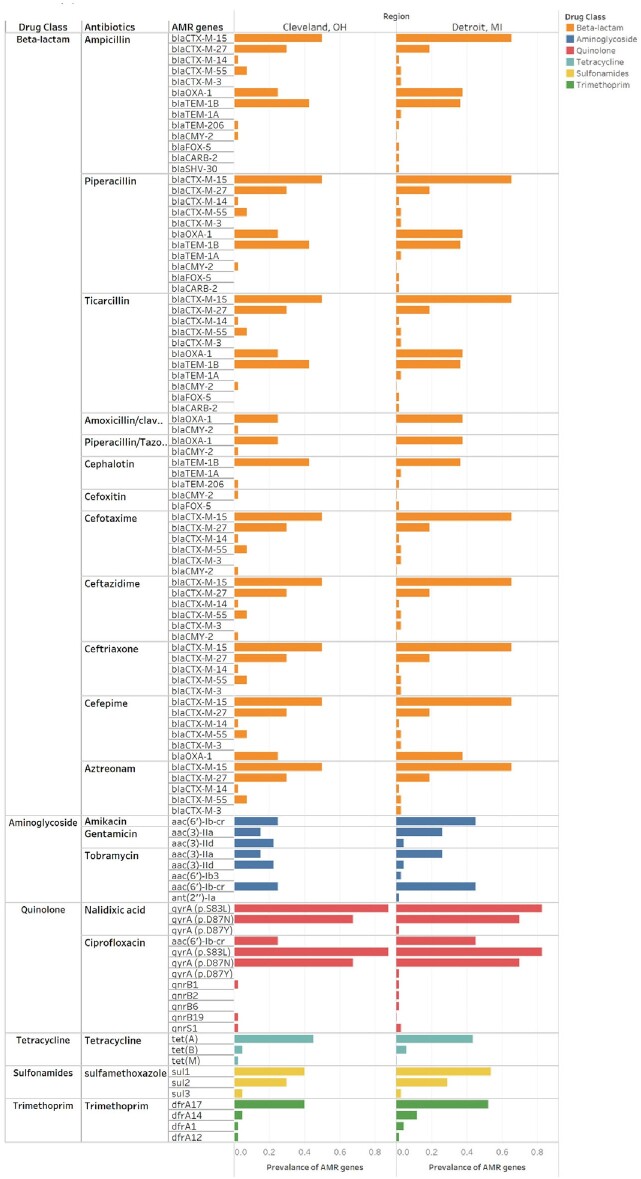
Prevalence of WGS identified AMR genes associated with antibiotics drugs.

**Conclusion:**

Most of the *E. coli* isolates contained various ESBL genes and their *gyrA* genes were mutated to quinolone resistant forms. Therefore, most of the isolates were resistant to many beta-lactam and quinolone drugs. WGS based prediction of phenotypic resistance showed good predictive values but needs more studies to increase sensitivity and specificity.

**Disclosures:**

**Keith S. Kaye, MD, MPH**, Abbvie: Advisor/Consultant|Abbvie: Honoraria|Entasis: Advisor/Consultant|Entasis: Honoraria|GSK: Advisor/Consultant|GSK: Honoraria|Merck: Advisor/Consultant|Merck: Honoraria|Shionogi: Advisor/Consultant|Shionogi: Honoraria|VenatoRx: Advisor/Consultant|VenatoRx: Honoraria

